# Effects of RAMEA-complexed polyunsaturated fatty acids on the response of human dendritic cells to inflammatory signals

**DOI:** 10.3762/bjoc.10.332

**Published:** 2014-12-30

**Authors:** Éva Rajnavölgyi, Renáta Laczik, Viktor Kun, Lajos Szente, Éva Fenyvesi

**Affiliations:** 1 Department of Immunology, University of Debrecen, Egyetem tér 1, Debrecen 4032, Hungary; 2 CycloLab Cyclodextrin Research & Development Laboratory Ltd., Illatos út 7, Budapest 1097, Hungary

**Keywords:** cell membrane, cell surface markers, cyclodextrin, enzyme immunoassay, flow cytometry, monocyte-derived dendritic cells, proinflammatory cytokines, RAMEA, solubilization

## Abstract

The *n*−3 fatty acids are not produced by mammals, although they are essential for hormone synthesis and maintenance of cell membrane structure and integrity. They have recently been shown to inhibit inflammatory reactions and also emerged as potential treatment options for inflammatory diseases, such as rheumatoid arthritis, asthma and inflammatory bowel diseases. Dendritic cells (DC) play a central role in the regulation of both innate and adaptive immunity and upon inflammatory signals they produce various soluble factors among them cytokines and chemokines that act as inflammatory or regulatory mediators. In this study we monitored the effects of α-linoleic acid, eicosapentaenoic acid and docosahexaenoic acid solubilized in a dimethyl sulfoxide (DMSO)/ethanol 1:1 mixture or as complexed by randomly methylated α-cyclodextrin (RAMEA) on the inflammatory response of human monocyte-derived dendritic cells (moDC). The use of RAMEA for enhancing aqueous solubility of *n*−3 fatty acids has the unambiguous advantage over applying RAMEB (the β-cyclodextrin analog), since there is no interaction with cell membrane cholesterol. In vitro differentiated moDC were left untreated or were stimulated by bacterial lipopolysaccharide and polyinosinic:polycytidylic acid, mimicking bacterial and viral infections, respectively. The response of unstimulated and activated moDC to *n*−3 fatty acid treatment was tested by measuring the cell surface expression of CD1a used as a phenotypic and CD83 as an activation marker of inflammatory moDC differentiation and activation by using flow cytometry. Monocyte-derived DC activation was also monitored by the secretion level of the pro- and anti-inflammatory cytokines IL-1β, TNF-α, IL-6, IL-10 and IL-12, respectively. We found that RAMEA-complexed *n*−3 fatty acids reduced the expression of CD1a protein in both LPS and Poly(I:C) stimulated moDC significantly, but most efficiently by eicosapentaenic acid, while no significant change in the expression of CD83 protein was observed. The production of IL-6 by LPS-activated moDC was also reduced significantly when eicosapentaenic acid was added as a RAMEA complex as compared to its DMSO-solubilized form or to the other two *n*−3 fatty acids either complexed or not. Based on these results *n*−3 fatty acids solubilized by RAMEA provide with a new tool for optimizing the anti-inflammatory effects of *n*−3 fatty acids exerted on human moDC and mediated through the GP120 receptor without interfering with the cell membrane structure.

## Introduction

The *n*−3 polyunsaturated fatty acids (omega-3 or *n*−3 PUFAs) contain multiple isolated carbon–carbon double bonds starting at the third carbon atom counting from the end of the molecule. The three main representatives involve α-linoleic acid (ALA, C18:3), eicosapentaenoic acid (EPA, C20:5) and docosahexaenoic acid (DHA, C22:6), which can be found in plants (flaxseed, walnut) and fish oil, respectively. ALA and linoleic acid (LA, C18:3 *n*−6 having the first double bond at the 6^th^ carbon atom from the chain end, therefore classified as *n*−6 PUFA) are essential fatty acids, because they cannot be synthesized by the human body. ALA can be converted to EPA and DHA [1], while LA is converted to arachidonic acid (AA, C20:4 *n*−6), the precursor of prostaglandins and related compounds playing crucial roles in inducing inflammatory reactions. EPA is the precursor of prostanoids with less inflammatory potential. DHA is a component of the phospholipid membranes found everywhere in the body [2]. Changes in the balance of *n*−3 and *n*−6 PUFAs in the diet upon low intake of *n*−3 PUFAs have been linked to several inflammation-related chronic diseases and certain mental illnesses [3].

Greenland Inuits with traditionally high seafood intake containing large quantities of *n*−3 PUFAs were found to show low morbidity rate from coronary heart disease [4]. Dietary supplementation with *n*−3 PUFA significantly decreased the risk of death after myocardial infarction [5]. Other studies also showed that *n*−3 fatty acids can change beneficially the blood lipid profile by decreasing the levels of triglyceride and very-low-density lipoprotein (VLDL) without reducing the level of low-density lipoprotein (LDL) [6]. Based on these data *n*−3 PUFAs have been considered as important contributors of the prevention and/or modulation of various human diseases [7].

Intake of dietary fats may influence inflammation in the gastrointestinal tract. In a long-term study conducted on 170 805 women a reduced risk of ulcerative colitis was observed for the participants with high intake of *n*−3 PUFAs [8]. The fatty acid composition of the diet, in particular, the proportion of different types of PUFAs, has an influence on the fatty acid composition of immune cells which is a chemical trigger for immune response, such as inflammation [9]. Supplementation of the diet of healthy human volunteers with *n*−3 PUFAs was unambiguously beneficial: the number of monocytes and the concentration of proinflammatory cytokines were reduced. Both animal experiments and clinical studies confirmed that fish oil supplementation was helpful in rheumatoid arthritis, inflammatory bowel disease, and certain types of asthma [5]. It was concluded that *n*−3 PUFAs in fish oil exhibit anti-inflammatory and immunomodulatory effects.

Recent studies also showed that the treatment of humans with ALA, EPA and/or DHA resulted in decreased expression of pro-inflammatory cytokines such as IL-1β, IL-6, TNF-α and were able to modulate DC to a direction that resulted in reduced cytotoxic T-cell responses and decreased level of inflammation. Furthermore, the secretion of the T-cell polarizing cytokines IL-10 and IL-12 was also modulated in a concentration-dependent manner [10–14].

In these studies PUFAs were solubilized in organic solvents, such as dimethyl sulfoxide (DMSO) with or without ethanol, because they could not be dissolved in water owing to their long alkyl chains. However, solubilization of PUFAs could also be achieved by aqueous cyclodextrin (CD) solutions, which allowed us to avoid organic solvents. CDs are cyclic oligosaccharides consisting of 6, 7 or 8 glucopyranose units (α-, β- and γ-CDs, respectively). They form a hollow cylindrical structure with a rather hydrophobic inner cavity, which readily adopts hydrophobic molecules forming inclusion complexes [15]. One of the most important effects of complexation is the enhanced solubility of poorly soluble compounds offering numerous possibilities for application especially in the development of improved pharmaceutical formulations [16]. The highly soluble CD derivatives, especially the methylated ones possess in most cases better solubilizing potential than their non-derivatized variants [17]. The solubilizing efficiency depends on the type, number and position of the substituents [18–19]. For example, water soluble palmitic acid and myristic acid complexed by RAMEB could be used for cultivation of otherwise non-cultivable leprosy-derived psychrophilic mycobacteria [20].

The methylated β-CDs are used as cholesterol depleting agents to disrupt caveolae and lipid rafts in the cell membrane as well as for replenishment of cholesterol in the form of water-soluble cholesterol/methyl β-CD complex to clarify the role of lipid rafts in various cellular processes [21–22]. For instance, DHA exerted anti-inflammatory effects via inhibition of the expression of cytokine-induced adhesion molecules in primary human retinal vascular endothelial (hRVE) cells, the target tissue affected by diabetic retinopathy [23]. To study whether the lipid rafts were involved in the mechanism of action, methylated β-CD was used for both the removal and the replenishment of cholesterol from cells treated by DHA. The methylated β-CD removes not only cholesterol but also some phospholipids from the membrane thus inhibiting the efflux function of P-glycoprotein [24–25], and at higher concentrations induces apoptosis in the rat alveolar cell line NR8383, in human alveolar basal epithelial lung A549 adenocarcinoma cells and in the T-cell leukemia cell Jurkat, respectively [26]. On the contrary, the methylated α-CD derivatives showed no interaction with membrane cholesterol and did not cause apoptosis in these cells [24].

The effect of methylated β-CD was also studied in immune cells and revealed the influence of cholesterol-content on the modulation of the T cell membrane, and also on the function of Jurkat cells as reviewed by Fülöp et al. [27]. Modulation of the cholesterol content in the cellular membrane with methylated β-CD seems to be an effective immunomodulatory intervention against immunosenescence with aging [28]. In other studies methylated β-CD used at high concentrations was found to inhibit the activation of lymphocytes while at low concentration (3–4 mM) it accelerated the proliferation of human peripheral blood mononuclear cells [29]. All these effects of methylated β-CD are based on the interaction with cholesterol.

**Figure 1:**
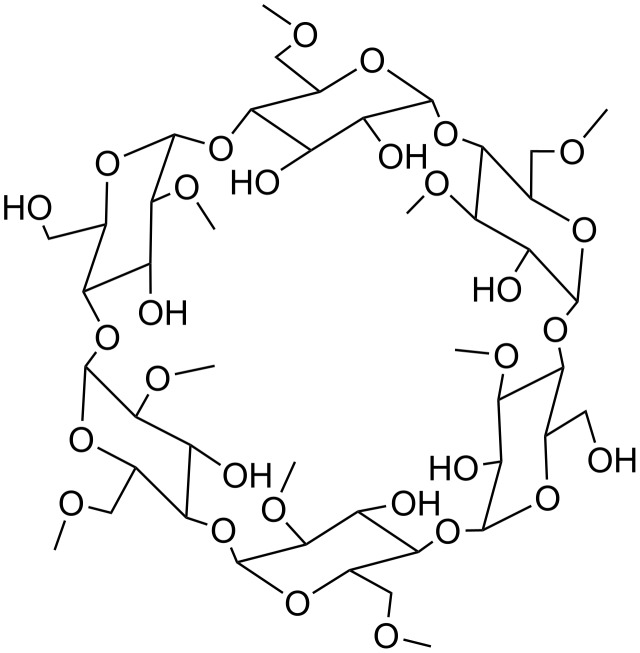
Structure of RAMEA.

**Figure 2:**
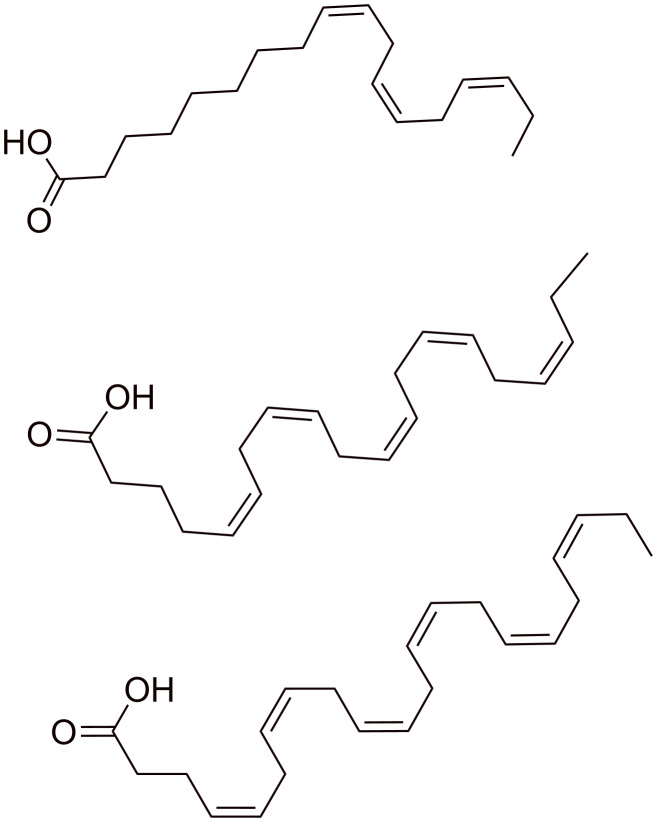
Structures of ALA, EPA and DHA.

The chemical structure of RAMEA is shown in Figure 1. The chemical structure of the *n*−3 PUFAs used for moDC treatments are shown in Figure 2.

The selection of human dendritic cells (DC) for monitoring the effects of *n*−3 PUFAs was based on our previous results demonstrating that the development of human monocyte-derived DC (moDC) is highly sensitive to environmental cues among them the level and composition of lipids and fatty acids [30]. These studies also revealed the phenotypic and functional heterogeneity of these cells depending on the actual tissue microenvironment that is able to drive monocyte differentiation either to inflammatory or to tolerogenic directions [31]. In the past decade it was also discovered that phylogenetically ancient Toll-like receptors (TLR) play essential roles in the recognition of conserved molecular patterns, which are able to alarm the innate immune system for fighting against invading pathogens and/or endogenous toxic or dangerous self molecules [32]. The capability of moDC with high phagocytic activity and the potential to translate the collected molecular information to other cell types lead to the notion that different subtypes and subsets of DC are central regulators of both innate and adaptive immunity and thus they can also be harnessed for vaccine development [33] and also for immunotherapeutic interventions [34]. By using high throughput approaches we also identified genes and signaling pathways [35], which govern diverse DC-associated functional activities such as mechanisms regulating antiviral immunity differently by the CD1a positive and CD1a negative moDC subsets [36]. Our recent studies focus to uncovering collaborative and/or opposing activities of TLR to gain insight to the inflammatory and regulatory networks governing moDC functions in the presence of various exogenous and/or endogenous signals [37]. In this context we identified distinct roles of human moDC types and subsets in supporting antiviral immunity [38–39], histamine-mediated modulation of moDC activities [40] and identified constraints of moDC functions under inflammatory conditions [41].

In the present study we aim to compare the effects of *n*−3 PUFAs on the phenotypic characteristics and the functional activities of human moDC activated by different inflammatory stimuli on day 5 of the moDC differentiation process. To compare the effects of ALA, EPA and DHA on moDC activities the cells were stimulated by bacterial lipopolysaccharide (LPS), the specific ligand of Toll-like receptor 4 (TLR4) and by synthetic polyinosinic:polycytidylic acid (Poly(I:C)) recognized by Toll-like receptor 3 (TLR3) specific for double stranded RNA (dsRNA). To avoid the interaction with cholesterol in the DC membrane in our in vitro experiments we used a non-cholesterol interacting CD, i.e., randomly methylated α-CD (RAMEA) for solubilization of *n*−3 PUFAs.

## Results and Discussion

### Solubilization of *n*−3 PUFAs

**Figure 3:**
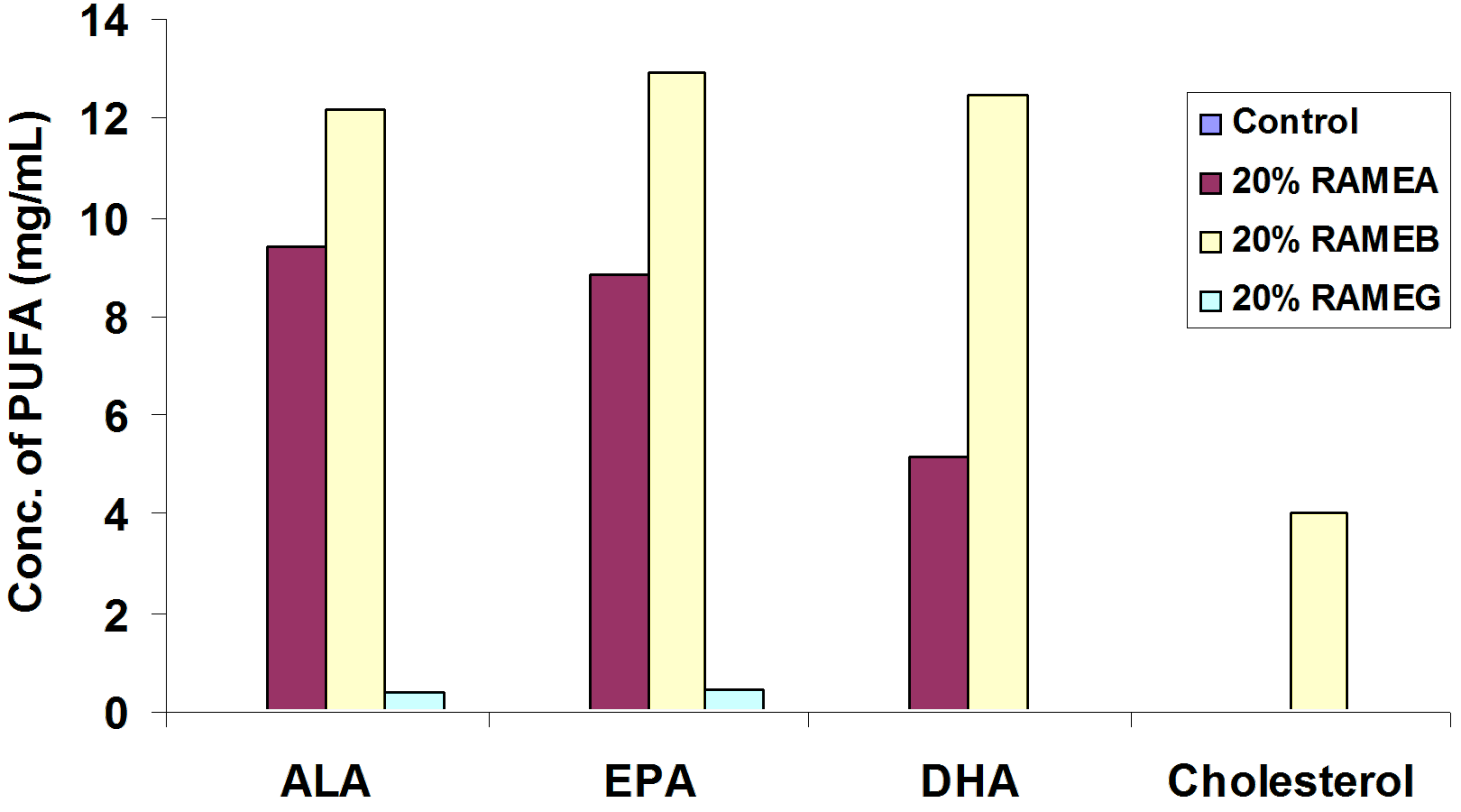
Solubility of *n*−3 PUFAs and cholesterol in water solutions of 20% randomly methylated α-, β- and γ-CDs (RAMEA, RAMEB and RAMEG) compared to distilled water (control).

Cyclodextrins (CDs) are efficient solubilizers of lipids [42]. The water-insoluble *n*−3 PUFAs were solubilized in aqueous solutions of randomly methylated α- and β-CD (RAMEA and RAMEB, respectively), but the similar γ-CD derivative, RAMEG was ineffective to modulate the solubility (Figure 3), while cholesterol could be dissolved by RAMEB only. Neither RAMEA nor RAMEG could solubilize cholesterol to a measurable extent. The solubilizing effect of RAMEA in the case of *n*−3 PUFAs seems to be high enough for using it as a solubilizer without the risk of interaction with cholesterol-rich lipid rafts in the cell membrane.

The complexation efficiency [43] calculated from the solubility data characterize the affinity of the various methylated CDs toward the lipids studied (Table 1). The complex association constants were not calculated because the intrinsic solubility in water is so low that it cannot be determined with a precision sufficient for the calculations.

**Table 1:** Complexation efficiency for fatty acid and cholesterol with randomly methylated CDs.

	ALA	EPA	DHA	Cholesterol

RAMEA	0.25	0.21	0.10	<0.001
RAMEB	0.43	0.41	0.35	0.08
RAMEG	0.012	0.012	<0.001	<0.001

The higher complexation efficiency corresponds to a higher affinity for complexation which is manifested in a higher solubilizing effect. Based on these results the inclusion complexes with 1–3% PUFA content were prepared by applying RAMEA. These complexes could readily be dissolved in water, and their aqueous solutions were used for all experiments performed with moDC.

### Effects of RAMEA-solubilized PUFAs on the expression of moDC cell surface proteins

Our previous results revealed that the expression of CD1a in moDC is controlled by the nuclear hormone receptor peroxysome proliferator activated receptor gamma (PPARγ) and its agonistic ligands are generated from poly-unsaturated fatty acids derived from modified LDL or acquired from exogenous sources assisted by ApoE [30]. We also found that the actual lipid and lipoprotein environment of the cell can result in the differentiation of two DC subsets identified by the expression level of the CD1 membrane protein referred to as CD1a^+^ and CD1a^−^ cells [31]. The continuous survey of the intracellular compartments of DCs is an important step in handling both protein and lipid antigens. However, it occurs differently for protein and glycolipid antigens as they use distinct intracellular transporter pathways. Furthermore, in contrast to other CD1 proteins, CD1a has an exceptionally short cytoplasmic tale, which is not associated with any adaptor molecule. Consequently, CD1a can reach the cell surface directly from the cytosol and also can acquire polar lipids from exogenous sources [44].

Considering that the most important biological function of the CD1a protein is the recognition, binding and presentation of glycolipids for innate immune cells [32], we hypothesized that upon interaction of lipids and fatty acids with various lipid binding receptors may also have the potential to influence the functional properties of moDC followed by internalization.

In our previous results CD1a^+^ moDC has been identified as an inflammatory cell type that is able to provoke inflammation, while its CD1a^−^ counterpart is characterized by the production of the anti-inflammatory cytokine IL-10 [33]. Based on these regulatory circuits the expression of CD1a emerged as a biomarker depending on the lipid and lipoprotein content of the environment.

We designed experiments where in vitro differentiated moDC were stimulated with LPS or Poly(I:C) mimicking bacterial and viral infections, respectively or were left untreated and the response of unstimulated and activated moDC was monitored by measuring the expression levels of CD1a and the DC activation marker CD83 by flow cytometry. CD83 was selected for monitoring DC activation based on its glycoprotein nature and the potential to act as both an activation and supression marker of DC depending on its monomeric or dimeric forms [45]. These functional attributes of CD83 offered us a potential tool for dissecting the protein and lectin mediated functions of CD83. However, our results demonstrated that PUFAs had no effect on the expression of CD83.

Changes in moDC phenotype related to cell activation was monitored and the secretion of pro- and anti-inflammatory cytokines that involved IL-1β, TNF-α, IL-6 and the T-cell polarizing cytokines IL-10 and IL-12, respectively was tested. The effects of the two solubilization methods (organic solvent and aqueous RAMEA solution) were also compared.

**Figure 4:**
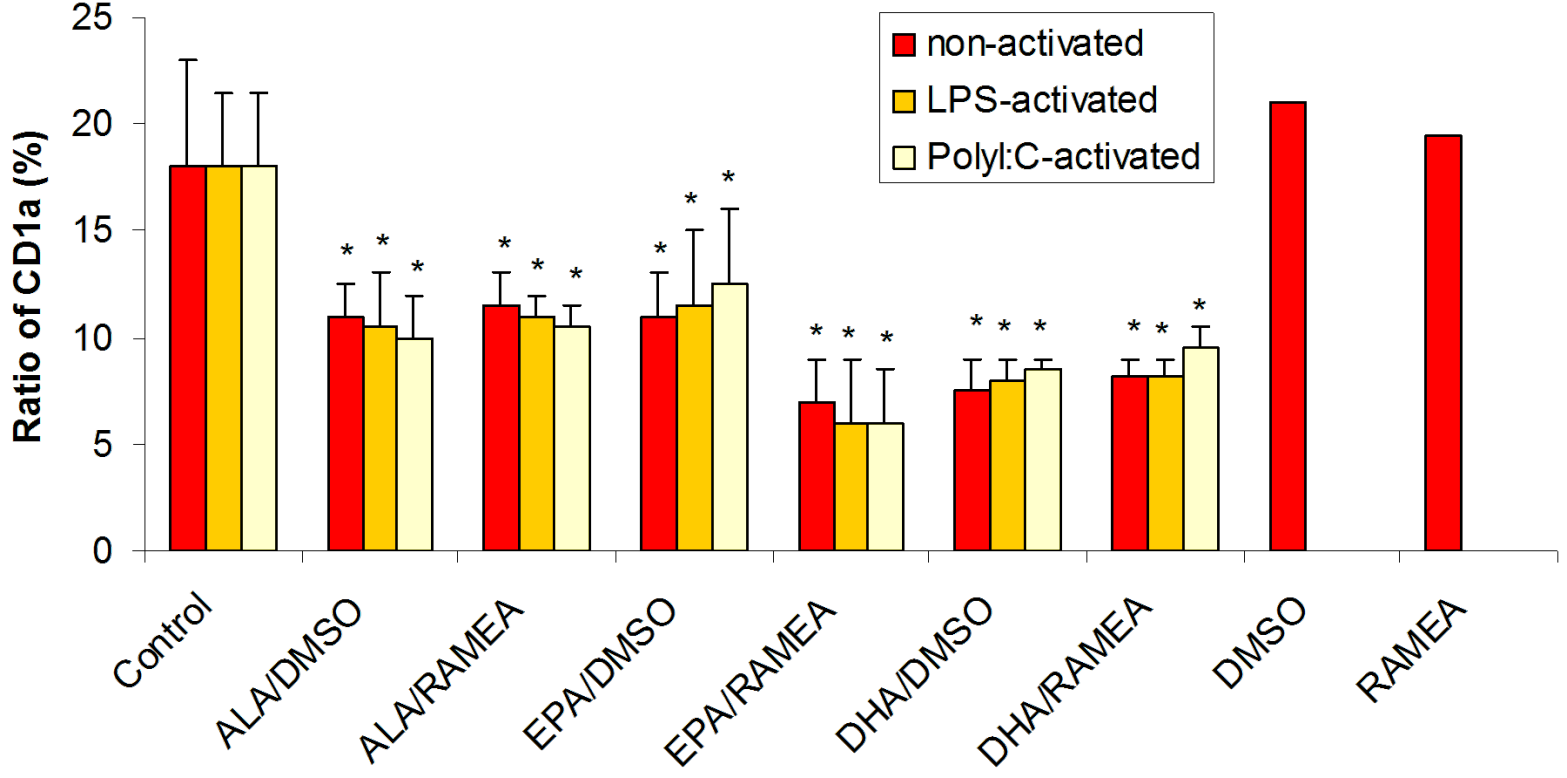
The effect of *n*−3 PUFAs on the expression of CD1a cell surface protein in resting (red), LPS-activated (yellow) and Poly(I:C)-activated (white) moDC (*N* = 6) (*p < 0.05 compared to the control).

According to the results shown in Figure 4 the ratio of resting CD1a^+^ cells did not change and remained in the range of 18–25% in the six individual donors tested, which could be further decreased by treatment with *n*−3 PUFAs independently on the mode of activation. Comparing the three *n*−3 PUFAs dissolved in DMSO/ethanol, DHA turned out to be more efficient in decreasing the proportion of inflammatory CD1a^+^ cells, than ALA or EPA. The solubilization method did not influence the effects of ALA and DHA, while the RAMEA-complexed EPA further reduced the expression of CD1a protein on the surface of moDC. The most efficient anti-inflammatory effect on the ratio of the CD1a^+^ DC subset was detected by the EPA/RAMEA complex. These results indicate that CD1a, acting as a lipid/glycolipid receptor on the cell surface may also be involved in the down modulation of CD1a expression by ALA, EPA and DHA. One possible mechanism of this effect could be the interference of *n*−3 PUFA with trafficking of CD1a proteins to the cell surface.

**Figure 5:**
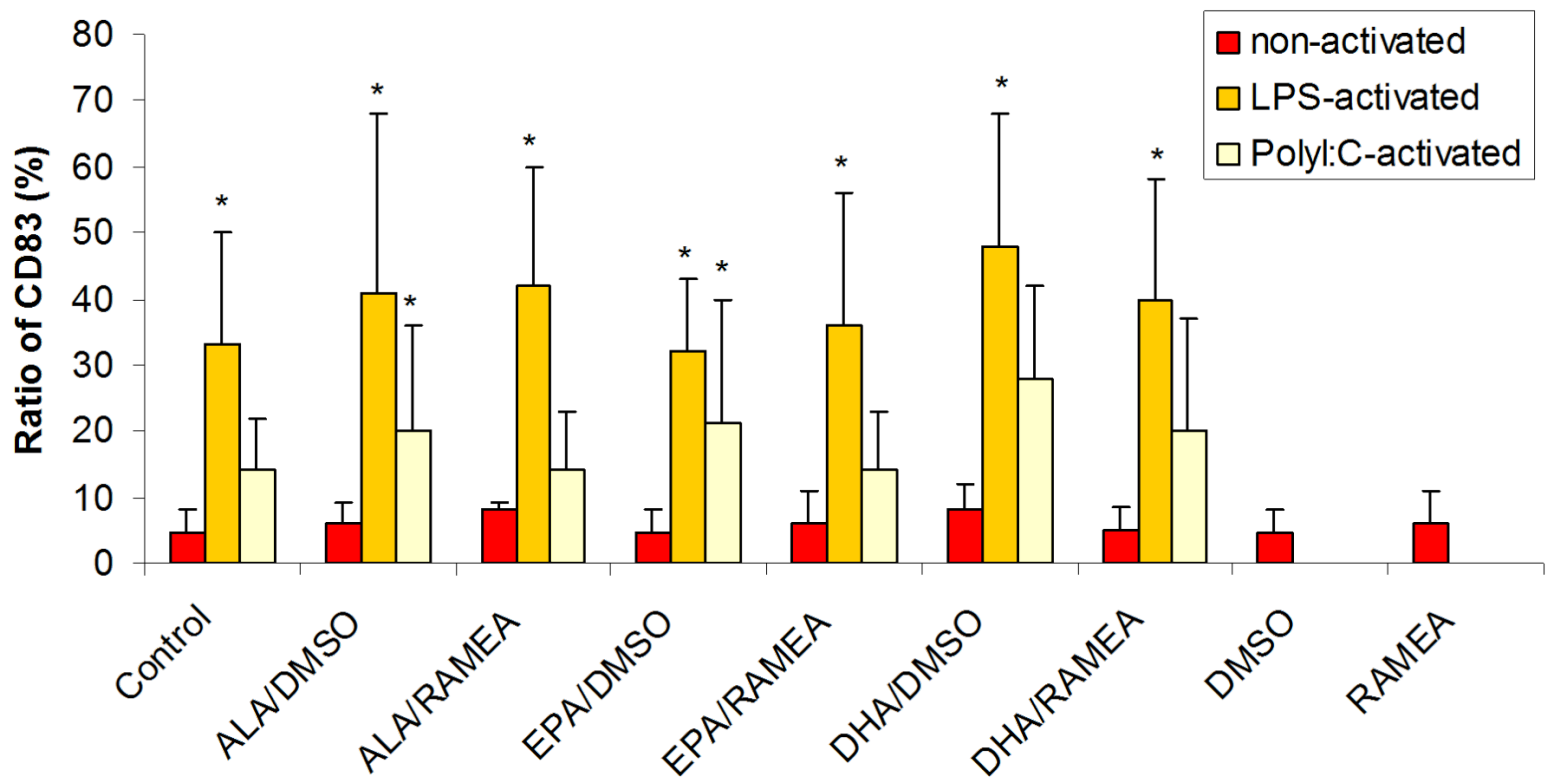
Effect of *n*−3 PUFAs on the expression of the CD83 activation marker in resting (red), LPS-activated (yellow) and Poly(I:C)-activated (white) moDC (*N* = 6) (*p < 0.05 compared to the control).

Activation of DC with both LPS and Poly(I:C), being the specific ligands of TLR4 and TLR3 receptors, respectively resulted in enhanced expression of the CD83 cell surface protein showing successful moDC activation (Figure 5). However, the treatment of DC with *n*−3 PUFAs had no significant impact on CD83 expression independent on the solubilizing method used. This result suggested that CD83 is not the direct target of the PUFA-mediated down modulatory effect.

**Figure 6:**
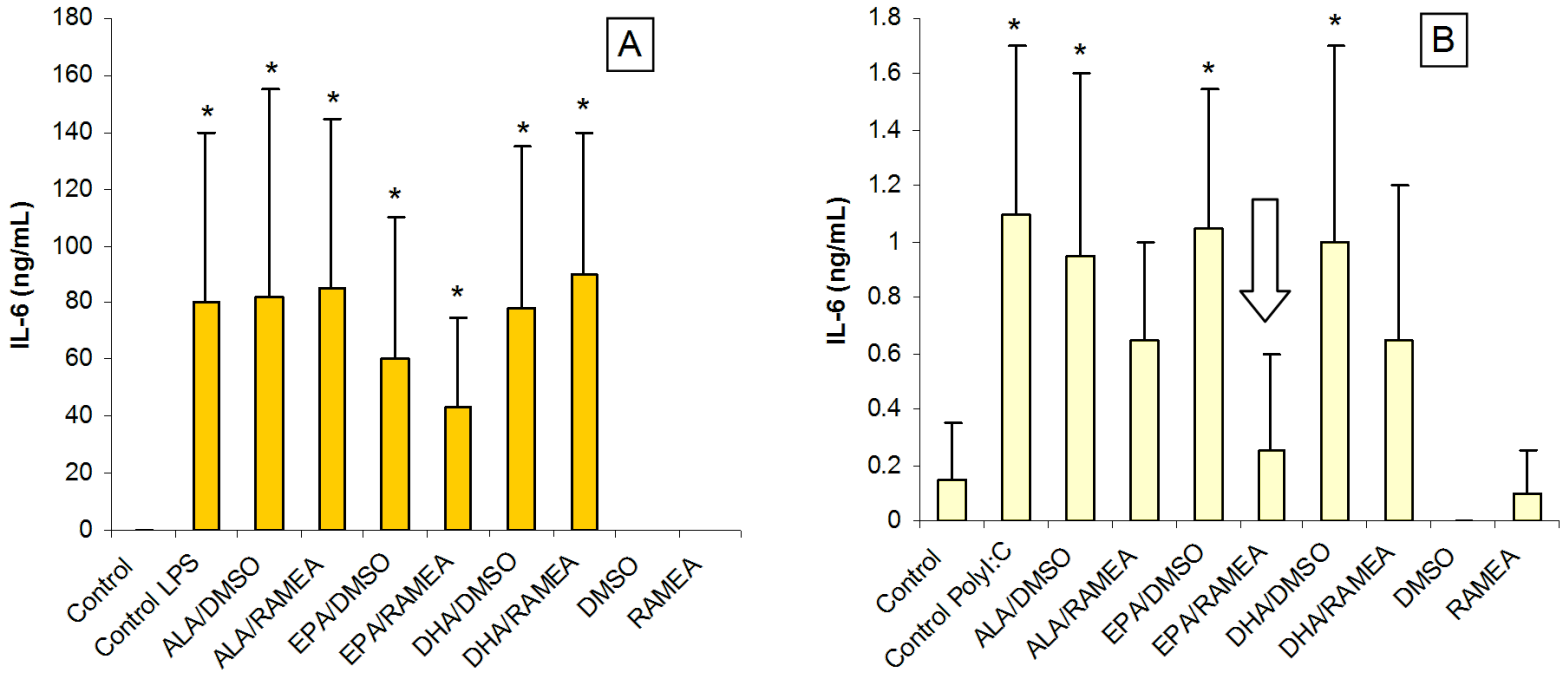
Effect of *n*−3 PUFAs on the expression of the pro-inflammatory cytokine IL-6 in moDC activated by LPS (A) or by Poly(I:C) (B) (*N* = 6) (*p < 0.05 compared to the control).

Earlier studies have shown that the anti-inflammatory effect of *n*−3 PUFAs is associated with the reduced production of pro-inflammatory cytokines both in mouse and human DCs [10–14]. In our experimental system the secretion of interleukin-1β (IL-1β), and tumor necrosis factor-α (TNF-α) was not influenced by the treatment of *n*−3 PUFAs even though the secretion of both cytokines could be enhanced markedly by both activators, i.e., LPS and Poly(I:C). In contrast, the level of IL-6 secreted by activated moDC was much higher than that of the non-activated cells, which hardly produced any IL-6. Activation of moDC by Poly(I:C) resulted in 1–2 orders of magnitude lower levels of IL-6 production than cells activated by LPS (Figure 6). The treatment with *n*−3 PUFAs did not cause significant changes in this pattern, except EPA complexed with RAMEA, which caused significant reduction in the level of Poly(I:C)-induced IL-6 secretion.

### Modulating the secretion of T-lymphocytes polarizing cytokines by *n*−3 PUFAs

The T-lymphocyte polarizing cytokines IL-12 and IL-10, produced by activated moDCs play a crucial role in the polarization of naïve CD4^+^ T-lymphocytes toward inflammatory or tolerogenic directions. Previous studies demonstrated that *n*−3 PUFAs have the potential to decrease the production of both cytokines [11–12]. To assess the effects of *n*−3 PUFAs on the expression of IL-10 and IL-12 cytokines we measured the expression levels of moDC-derived IL-10 and IL-12 by ELISA. However, the treatment of PUFAs in our hands did not cause such a decrease in cytokine secretion, although the secretion of both cytokines was increased upon activation by LPS or Poly(I:C), and no difference between the cells treated with any of the 3 fatty acids could be detected (data not shown).

Similar results were obtained when moDC-induced T-cell polarization was studied by the ELISPOT assay monitored by the response of moDC-induced autologous T-lymphocytes measured by IFNγ and IL-17 cytokine secretion. Interestingly, we observed that the number of IL-17-producing T-cells induced by Poly(I:C)-stimulated moDC gave rise to even lower number of IL-17 producing cells than produced by non-activated or LPS-activated moDC suggesting that the down modulation of moDC stimulation by *n*−3 PUFAs could be translated to T-lymphocytes as an inhibitory/tolerogenic signal. Based on these results we suggest that *n*−3 PUFAs act at the level of innate immunity via down modulating selected functional activities of inflammatory moDCs, which also have the capability to modify effector T-lymphocyte responses.

### Effects of *n*−3 PUFAs on the expression of the GPR120 receptor

G-protein-coupled receptor-120 is a membrane bound receptor with 7 trans-membrane domains, which bind specifically to long chain fatty acids. The ligands of GPR120 involve multi-unsaturated long-chain fatty acids present in the outer membrane of adipocytes, monocytes and dendritic cells. In contrast to small chain fatty acids they play a role in the regulation of body weight and glucose homeostasis, and when activated by omega-3 fatty acids, they support anti-inflammatory effects by inhibiting inflammatory cytokine secretion via signaling through the GPR120 receptor This occurs with the assistance of the adapter protein β-arrestin-2 that directly associates with GPR120 [46].

**Figure 7:**
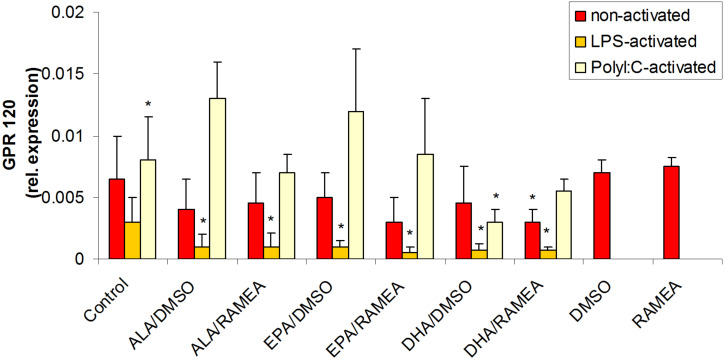
The effect of *n*−3 PUFAs on the expression of GPR120 receptor in resting (red), LPS-activated (yellow) and Poly(I:C)-activated (white) moDC (*N* = 6) (*p < 0.05 compared to the control).

To study the role of the GPR120 receptor in the regulation of pro-inflammatory cytokine production we tested the immunomodulatory effects on DCs by monitoring its expression levels in resting cells as compared to cells activated by LPS or Poly(I:C). Our results showed that LPS-induced inflammation caused decreased GPR120 expression and the treatment with *n*−3 PUFAs further reduced the expression of this receptor indicating consumption of the receptor induced preferentially by DHA (Figure 7). Most importantly, the RAMEA-solubilized EPA was also efficient to mediate this modulatory effect, while Pol(I:C) exerted an adverse effect and resulted in slightly elevated GPR120 expression indicating the involvement of different regulatory pathway in LPS- and Poly(I:C)-induced moDC modulation.

## Conclusion

The *n*−3 PUFAs can be solubilized by aqueous RAMEA solutions similarly to RAMEB without the risk of cholesterol removal from the cell membrane. This solubilization method avoids the use of organic solvents, which may be toxic for differentiating and functional cells. The response of moDCs to PUFA treatments induced by inflammatory stimuli and mimicking bacterial and viral infections demonstrated that the phenotype of activated moDCs could be modified and the secretion of pro-inflammatory cytokines could be decreased as a result of PUFA treatment. The anti-inflammatory effect of the *n*−3 PUFAs was also demonstrated by the decreased expression of CD1a membrane protein in the cell surface, the decreased secretion of the pro-inflammatory cytokine IL-6 and the PUFA-mediated modulation of GPR120 receptor expression dependent on the mode of moDC activation. The treatment of moDCs with *n*−3 PUFAs showed no significant changes in the expression of the CD83 activation marker, in the secretion of IL-1β and TNF-α cytokine secretion, and in the production of moDC-mediated IFNγ and IL-17 cytokine production involved in T-lymphocyte proliferation and activation. These results altogether indicate the delicate regulation of PUFA-induced effects on DC functional activities ultimately leading to a balancing effect of inflammatory processes.

Comparison of the three fatty acids studied showed that they could cause similar changes in most functions tested. Comparison of the two solubilizing methods (organic solvent and aqueous cyclodextrin solution) revealed that RAMEA-solubilized EPA was more effective in reducing the expression of CD1a, the secretion of IL-6 and the down regulation of GPR120 receptor expression than EPA dissolved in DMSO/ethanol. Based on these results *n*−3 PUFAs solubilized by RAMEA provide with a new tool for optimizing the anti-inflammatory effects exerted on human moDC and mediated via the GPR120 receptor without interfering with the cell membrane structure.

## Experimental

### Fatty acids, cyclodextrins

The fatty acids were purchased from Sigma-Aldrich. The randomly methylated β-cyclodextrin (RAMEB, the number of methyl groups/CD (DS) is 12) is the product of Wacker Chemie, Munich, Germany, while the α- and γ-CD analogues with DS 8.7 and 12.6 were produced by CycloLab, Hungary.

#### Solubility measurement

The solubility of fatty acids in CD solutions was determined by adding PUFAs to the aqueous solutions at 20% RAMEA, RAMEB or RAMEG, respectively, stirring for 24 h at rt and then analyzed the filtered saturated solutions by HPLC using Hypersil ODS column (4 × 125 mm, particle size 5 μm) with guard column, mobile phase: acetonitrile/water 75:25 with 0.15% concentrated phosphoric acid, 2.0 mL/min flow rate, at 40 °C and UV detection at 210 nm.

The PUFA/RAMEA complexes were prepared by lyophilization of the filtered, saturated solutions. The fatty acid content of the lyophilized complexes was 2.3%, 3.1% and 1.4% for ALA, EPA and DHA complexes, respectively.

#### Isolation of primary human monocytes and differentiation of monocyte-derived dendritic cells

Peripheral blood mononuclear cells (PBMC) were separated from buffy coats by Ficoll gradient centrifugation (Amersham Biosciences, Uppsala, Sweden). Monocytes were isolated by positive selection using magnetic cell separation with anti-CD14-conjugated microbeads (Miltenyi Biotech, Bergisch Gladbach, Germany). The proportion of CD14^+^ cells was determined by flow cytometry and was found to be 96–99% dependent on the blood donor. The purified cells were cultured at a density of 10^6^ cells/mL in serum free AIMV medium supplemented with 80 ng/mL granulocyte-macrophage colony stimulating factor (GM-CSF) purchased from Gentaur and 50 ng/mL IL-4 from Peprotech.

#### PUFA treatment of moDC

On day 0 and day 2 of the in vitro moDC differentiation process the cultures were supplemented with GM-CSF and IL-4 and were treated with 20 µM/mL *n*−3 PUFAs. On day 5 the fully differentiated cells were activated by 500 ng/mL bacterial lipopolysaccharide (LPS) (Sigma Aldrich) or by 500 ng/mL Poly(I:C) (InvivoGen, San Diego, CA, USA) or were left untreated. The negative controls were treated by DMSO or by 20 µM/mL RAMEA. The positive controls were stimulated by LPS or Poly(I:C) in the absence of PUFAs.

#### Cytokine measurements

Supernatants of the activated moDC were collected on day 6 of the differentiation process and the concentration of secreted cytokines was determined by cytokine-specific OptEIA Set^®^. Determination of cytokine levels was performed with StepOnePlus equipment (AB-Applied Biosystems) and was evaluated with the help of the StepOne Software v2.1 and Excel.

#### Flow cytometry

Phenotyping of moDC was performed by flow cytometry using anti-CD83-PE and anti-CD1a-FITC specific antibodes from BioLegend at the protein level (San Diego, CA, USA). Fluorescence intensities were measured with FACSCalibur (BD Biosciences). Data analysis was performed with the FlowJo software (Tree Star, Ashland, OR, USA).

#### Determination of GPR120 expression levels

GPR120 expression levels were measured by real-time quantitative PCR (Q-PCR). Total RNA was extracted using TRI Reagent (Molecular Research Center, Inc., Cincinnati, OH, USA) and was reverse-transcribed using the High Capacity cDNA RT Kit of Applied Biosystems (Carlsbad CA). The GPR120 gene expression assays were purchased from Applied Biosystems. Results were normalized to the housekeeping gene cyclophilin (Integrated DNA Technologies, Coralville, IA, USA). Q-PCR was performed using the ABI StepOne Real Time PCR System (Applied Biosystems) and cycle threshold values were determined by using the StepOne v2.1 Software (Applied Biosystems).

#### Statistical analysis

The results of flow cytometry, Q-PCR and ELISA studies were analyzed by one-way ANOVA with Bonferroni post-hoc test using the GraphPad Prism v.6 software (GraphPad Software Inc., La Jolla, CA, USA). Differences were considered to be statistically significant at *p* < 0.05.
